# Analysis of Prairie Soil

**Published:** 1851-03

**Authors:** 


### ARTICLE IX.

### *Analysis of Prairie Soil.*

Although not strictly professional in its character, the following paper, taken from the U. S. Patent Office Report, for 1849-50, will be found of interest, especially to those who are studying the influence of the soil upon the health in different localities. E.

(We insert with pleasure the following analysis of a specimen of prairie soil, from the farm of Dr. J. A. Kennicott, of The Grove, Ill., made by Prof. James V. Z. Blaney; of Chicago.)

The soil analyzed was taken from a "roll" of the prairie, at or near the summit of the "roll" or elevation. A sod two inches thick was first removed, and the specimen then taken up with a spade, about 6 inches deep; the central portion of which was used for the analysis.

*Description.*—The soil was a loose friable loam, of a very intense black color when moist, greyish-black when dry. It contained some small fibres of grass roots, and no gravel. *A Mechanical Analysis* was first made, as follows: A weighed portion of the soil was treated with distilled water, and agitated for some time; then allowed to settle for one minute; the water poured off into another vessel, and allowed to stand to deposit the pulverulent matter which it held in suspension. This process was continued until the washings ceased to hold any particles in suspension. The sandy matter left, and the pulverulent matter washed over were each collected on a weighed filter, washed, dried at 212° F., and weighed. The filtrates of both were mixed, evaporated to dryness at 212°, and weighed, giving matter soluble in cold water. A separate portion of the soil, which had been exposed for a long time to a moderately dry air, was dried at 212°, and the hygrometric moisture thus ascertained. These processes gave the following

*Results of the Mechanical Analysis.*

| | Per. cent. |
|---|---|
| Hygrometric moisture, | 3.50 |
| Sandy particles, | 66.90 |
| Finely divided do. | 29.20 |
| Matter soluble in cold water, | 0.10 |
| | 99.70 |
| Loss | 0.30 |
| | 100.00 |

A separate analysis was made of the coarser and finer particles. Analysis of the coarser particles gave the following results:

| | Per. cent. |
|---|---|
| Organic matter and combined water, together, | 8.00 |
| Matter insoluble in hydrochloric acid, say, silica, | 84.00 |
| Combined silica (probably in combination with alkalies) | 0.50 |
| Alumina, | 2.50 |
| Sesquioxide of iron, | 3.00 |
| Carbonate of lime, | 1.00 |
| Magnesia and alkalies together, | 2.00 |
| | 101.00 |

Analysis of the finely divided particles gave

| | Per. cent. |
|---|---|
| Organic matter and combined water, | 10.00 |
| Matter insoluble in hydrochloric acid, say, silica, | 76.00 |
| Combined silica, (a trace) | |
| Alumina, | 4.25 |
| Sesquioxide of iron, | 4.25 |
| Carbonate of lime, | 1.50 |
| Magnesia and alkalies together, | 2.50 |
| | 98.50 |

The error in the former analysis is, I think, in a measure, due to the fact, that the iron exists in the sandy matter as a protoxide, probably in combination with silica; hence, when calculated as a sesquioxide, it gives too much by about 0.40 per cent.

In the latter analysis, I presume that the iron exists in the form of a protocarbonate. If such be the case, nearly two per cent should be added, to obtain a correct result.

The analysis of the soil as a whole, as calculated from the above, would give the following as a very close approximation to the truth:

| | |
|---|---|
| Hygrometric water, | 3.500 |
| Organic matter and combined water, | 8.166 |
| Silicious matter insoluble in hydrochloric acid | 77.286 |
| Silica combined, | 0.328 |
| Alumina, | 2.880 |
| Sesquioxide of iron, | 3.458 |
| Carbonate of lime, | 1.094 |
| Matter soluble in cold water, (not examined) | 1.000 |
| Magnesia and alkalies, (not separated) | 2.369 |
| | 100,081 |

Had a little more time been allowed to me, I would have ascertained with accuracy the amount of alkalies, and also in what state of combination they exist, as carbonates, sulphates, or silicates. I would also have critically examined the organic matter, and tested for the presence of phosphates. I have ascertained that nitrogen is present in the organic matter, as ammonia is evolved at a high temperature.

For the absorbent power of the soil, I find that a specimen, which had been exposed for some time to dry air, when exposed 48 hours over water at 60° F., gains 3 per cent. of water by capillary absorption. Another portion, of the same degree of dryness, exposed *in vacuo* over sulphuric acid at 60° F., lost, in 48 hours, 3.20 per cent. of water, by evaporation into a perfectly dry atmosphere.

The difference in the amount of water retained by capillary attraction, in an atmosphere saturated at 60° F., and that in perfectly dry air at the same temperature, may be stated at 6.20 per cent.

